# Mesenchymal stromal cells promote the drug resistance of gastrointestinal stromal tumors by activating the PI3K-AKT pathway via TGF-β2

**DOI:** 10.1186/s12967-023-04063-0

**Published:** 2023-03-25

**Authors:** Yu Zhao, Zuyi Weng, Xuan Zhou, Zhi Xu, Bei Cao, Bin Wang, Juan Li

**Affiliations:** 1 Phase I Clinical Trials Unit, The Affiliated Drum Tower Hospital of Nanjing University Medical School, Nanjing, 210000 China; 2 Clinical Stem Cell Center, The Affiliated Drum Tower Hospital of Nanjing University Medical School, Nanjing, 210000 China

**Keywords:** Mesenchymal stromal cells, Tumor microenvironment, Gastrointestinal stromal tumors, TGF-β, PI3K-AKT, Imatinib mesylate, Drug resistance

## Abstract

**Background:**

Gastrointestinal stromal tumors (GISTs) are the prevailing sarcomas of the gastrointestinal tract. Tyrosine kinase inhibitors (TKIs) therapy, exemplified by Imatinib mesylate (IM), constitutes the established adjuvant therapy for GISTs. Nevertheless, post-treatment resistance poses a challenge that all patients must confront. The presence of tumor heterogeneity and secondary mutation mechanisms fail to account for some instances of acquired drug resistance. Certain investigations suggest a strong association between tumor drug resistance and mesenchymal stromal cells (MSC) in the tumor microenvironment, but the underlying mechanism remains obscure. Scarce research has explored the connection between GIST drug resistance and the tumor microenvironment, as well as the corresponding mechanism.

**Methods:**

Immunofluorescence and fluorescence-activated cell sorting (FACS) methodologies were employed to detect the presence of MSC in GIST samples. The investigation encompassed the examination of MSC migration towards tumor tissue and the impact of MSC on the survival of GIST cells under IM treatment. Through ELISA, western blotting, and flow cytometry analyses, it was confirmed that Transforming Growth Factor Beta 2 (TGF-β2) triggers the activation of the PI3K-AKT pathway by MSC, thereby facilitating drug resistance in GIST.

**Results:**

Our findings revealed a positive correlation between a high proportion of MSC and both GIST resistance and a poor prognosis. In vitro studies demonstrated the ability of MSC to migrate towards GIST. Additionally, MSC were observed to secrete TGF-β2, consequently activating the PI3K-AKT pathway and augmenting GIST resistance.

**Conclusions:**

Our investigation has revealed that MSC within GISTs possess the capacity to augment drug resistance, thereby highlighting their novel mechanism and offering a promising target for intervention in GIST therapy.

**Supplementary Information:**

The online version contains supplementary material available at 10.1186/s12967-023-04063-0.

## Introduction

Gastrointestinal stromal tumors (GISTs), the most prevalent sarcomas affecting the gastrointestinal tract, were previously associated with an unfavorable prognosis. Approximately 70–80% of GISTs harbor a mutation in the KIT (CD117) or platelet-derived growth factor receptor-alpha (PDGFRA) genes. Prior to treatment with tyrosine kinase inhibitors (TKIs), GISTs were known to exhibit a high level of resistance towards conventional chemotherapy and radiotherapy [1]. Although surgical resection has been the cornerstone of GIST therapy, the recurrence of GISTs after surgery is a common occurrence within a short period of time.

Since 2002, imatinib mesylate (IM), a tyrosine kinase inhibitor that targets KIT and PDGFRA in GIST, has emerged as the gold standard therapy for metastatic and unresectable GIST. IM treatment elicits sustained responses or disease stabilization in approximately 85% of GIST patients [2, 3]. However, the majority of GIST patients will inevitably develop resistance to IM treatment, resulting in recurrence or metastasis [4]. The underlying mechanisms responsible for IM resistance have yet to be fully elucidated. Several studies have suggested that primary TKI resistance, defined as progression within the first 6 months of therapy, may be attributed to PDGFRA p.D842V substitution or KIT exon 9 duplications [5]. Additionally, secondary mutations occurring in KIT/PDGFRA have been linked with secondary resistance [6–8].

The tumor microenvironment (TME) encompasses a complex milieu of stromal cell types, such as MSC, cancer-associated fibroblasts (CAFs), the extracellular matrix (ECM), immune cells, and endothelial cells. TME plays a pivotal role in the initiation, progression, and prognosis of various sarcomas [9]. Increasing evidence suggests that TME is vital in developing drug resistance in tumor cells. MSC are a heterogeneous population of multipotent adult cells found in many tissues. They could be continuously recruited and become integral components of TME [10, 11]. Upon integration into the TME and subsequent interaction with tumor cells, MSC are known to exert influence on multiple cancer hallmarks, such as the development of chemotherapy resistance [12, 13]. Remarkably, it has been observed that the interplay between bone marrow stromal cells and leukemia cells can play a significant role in conferring resistance to TKI therapy *in *ex vivo [14].

Hence, it is postulated that MSC could migrate to TME and contribute to TKI therapy resistance in GIST. Our present findings indicate a significant association between MSC proportion, tumor drug resistance, and GIST prognosis. It has been suggested that MSC may activate their PI3K-AKT-mTOR signaling pathway through paracrine secretion of TGF-2, thereby promoting the drug resistance of GIST.

## Materials and methods

### Clinical features of GIST patients

We obtained paraffin-embedded biospecimens from 12 individuals who underwent surgical resection for GIST. Paired samples from each patient comprised one sample prior to targeted treatment, demonstrating sensitivity to Imatinib, and one sample post-resistance development. Pathological assessment verified the absence of common drug resistance mutations in all post-resistance samples.

Fifty additional patients with GIST were included in the study, with complete follow-up data available, including progression-free survival and overall survival. Their surgical resection specimens were utilized to explore the correlation between MSC and clinical outcomes.

The diagnosis of GIST was confirmed by the Pathology Department of Nanjing Drum tower Hospital in accordance with the criteria established by the World Health Organization.

### Cell culture

Human bone marrow-derived MSC (BMMSC) was provided by the Center for Clinic Stem Cell of Nanjing Drum Tower Hospital. BMMSC were found to express CD73, CD90, CD105, and CD166, while lacking expression of CD34, CD45, and HLA-DR, and demonstrated potential for trilineage differentiation, as shown in Additional file 1: Fig. S2. GIST-882 and GIST-T1 cell lines were procured from ATCC (Manassas, VA, USA) and were cultured using L-DMEM supplemented with 12% fetal bovine serum, 2 mM L-glutamine, and 100 μg/ml streptomycin (Cat No. 11885084, Life Technologies, USA). Cells were passaged at a ratio of 1:3 when 70% confluent. Only MSC in passages 2 to 3 were used for all experiments. To inhibit GIST cell growth or induce apoptosis in vitro, 50 nM Imatinib was added to the medium.

### Identification and enumeration of MSC cells in tumor tissues

To identify markers of MSC in GIST tissues and their ability to trilineage differentiation, we first prepared fresh GIST surgical samples into single cell suspensions. Tissues were cut within 1 h of dissection and digested at 37 °C with collagenase type I, collagenase type III, and hyaluronidase in L-DMEM containing 12% FBS for half an hour with agitation. Thereafter, the supernatant was passed through a series of 40 µm nylon mesh cell filters and then separated into a new tube. Centrifuge at 900 g for 5 min and collect the precipitate. The precipitate was combined and washed twice in serum-free L-DMEM. The MSC cells were then initially purified using CD90-positive Magnetic Activated Cell Sorting (MACS). The obtained cells were cultured in complete MSC medium consisting of L-DMEM, 12% fetal bovine serum (Cat No. A3840202, Gibco, USA), 1-mM L-glutamine and 1X streptomycin (Cat No. 11885084, Life Technologies, USA). After 24 h, the cells were washed twice with PBS to remove non-adherent cells and incubated until they reached confluence. Thereafter, cells were trypsinized (0.25% trypsin and 0.1% ethylene diamine tetraacetic acid) and subcultured at a density of 5000 cells/cm^2^. Cells obtained were assessed for several mesenchymal features including adhesion to plastic, trilineage differentiation, and the presence of typical mesenchymal stem cell surface markers (CD166, CD105, CD90, and CD73 were positive; CD45, CD34, and HLA-DR were negative).

The samples used to count MSC in tumor tissues using fluorescence-activated cell sorting (FACS) are paraffin-embedded clinical surgical samples. The samples were prepared into single-cell suspensions by the following steps: Paraffin sections of 20 μm thickness were first de-paraffinised by immersion in xylene for 15 min and then rehydrated by transferring to 100%, 95%, and 70% alcohol for 5 min respectively in sequence. The antigen was then repaired by heating in citrate buffer at 100 °C for 30 min. The tissue was then incubated for 1 h at 37 °C using collagenase type I, collagenase type III, and hyaluronidase to disintegrate the tissue. The cell suspension is then filtered through a 40-μm cell strainer to remove any remaining tissue debris. Centrifuge the filtered cell suspension at 300 × *g* for 10 min to pellet the cells. Cells were resuspended and washed twice with PBS. The obtained single cell suspensions were stained using the MSC Characterization Antibody Panel (Cat No. 1000354, STEMCELL Technologies, Canada), counted by flow cytometry, and the proportion of MSC cells in the total cell count was calculated.

### MSC's trilineage differentiation protocol

MSC cells from tumor tissue or human bone marrow sources were cultured in a complete DMEM medium as described above. The third-generation MSC were trypsinized to prepare a cell suspension at a concentration of 1 × 10^5^ cells/ml and seeded in 6-well plates. When the cells reach 60–70% confluence, they are ready for trilineage differentiation.

According to the manufacturer's recommended protocol, MSC cells were differentiated into osteoblasts, adipocytes, and chondrocytes using a specific osteogenic, lipogenic, or chondrogenic induction and maintenance medium (Lonza, Walkersville, MA). After 21 days of continuous culture, differentiated MSC cells are ready for assay. To analyze the results of osteogenic differentiation, the cells were stained with 40 mM Alizarin Red. Calcium deposits were visible within the osteoblast spheres. To analyze the results of adipogenic differentiation, the cells were stained with Oil Red O. For chondrogenic differentiation, pelleted specimens were fixed in formalin, embedded in paraffin, and thinly sectioned and stained for glycosaminoglycans using Safranin O stain.

### Co-culture of MSC with GIST cells

In the contact co-culture system, MSC and GIST cells are cultured together in the same dish. MSC were inoculated at a concentration of 1 × 10^4^ cells/ml in flasks and after 24 h, GIST cells were inoculated in the same flasks at a concentration of 1 × 10^5^ cells/ml. The culture medium used was L-DMEM containing 12% fetal bovine serum, 1 mM L-glutamine, and 100 μg/ml streptomycin (Cat No. 11885084, Life Technologies, USA). The cells were then incubated at 37 ℃ in a humidified atmosphere with 5% CO_2_.

The 6-well plate is equipped with a transwell apparatus in each well, where MSC and GIST cells are situated inside and outside the chamber, respectively. These cells do not make physical contact with one another, and the medium is interchangeable. The cell density and medium composition are consistent with those of the contact co-culture model.

### Preparation of conditioned media

In order to obtain conditioned media (CM), MSC and GIST cells were co-cultured in either the contact co-culture or non-contact co-culture system, as described previously. The contact co-culture method yielded contact conditioned medium (contact CM), while transwell conditioned medium (transwell CM) was obtained using the non-contact co-culture system. Additionally, GIST-CM and MSC-CM were obtained by separately culturing GIST and MSC cells. All CMs were collected every 6 h, centrifuged, and filtered using a vacuum-driven cup apparatus (Millipore Express PLUS, Merck Millipore, Germany), and then stored in a frozen state. Prior to use, the CMs were thawed and mixed with complete culture media at a 10–20% ratio.

### Cell proliferation assay

Cell proliferation was evaluated by utilizing the CCK-8 (Cat No. CK04, Dojindo, Japan) assay, following the manufacturer's guidelines. In brief, 5 × 10^3^ GIST-882 or GIST-T1 cells were seeded in 96-well plates and then incubated with MSC-CM or control media after a 6-h starvation period. Each group had 3 duplicates. Following 48 h of incubation, 10 μl of CCK-8 solution was added to each well, followed by an additional 2-h culture at 37 °C. Optical density was measured at 450 nm using an automated cell counter (Nexcelom, cellometer Mini, USA).

### Transwell migration assay

The migration of human BMMSC towards GIST cells was investigated using the Transwell assay. In brief, 5 × 10^4^ human BMMSC in 300 μl of control media were seeded in the top chamber of each Transwell (Cat No. 3401, Corning, NY, USA). In the bottom chamber, 700 μL of control medium supplemented with 10% or 20% GIST-CM was added. After a 36-h incubation period, the membranes were stained with crystal violet solution and paraformaldehyde. The cells on the upper surface of the filter were removed, and the cells that had migrated through the inserts' membrane were observed and quantified using Image J software (version 1.4.3.67) under a light microscope (Leica microscope, USA). Three images were captured for each membrane, and the experiment was performed in triplicate.

### Wound-healing migration and Transwell invasion analysis

For the wound-healing migration experiment, GIST-882 cells were collected, planted in triplicate wells of a 6-well plate, and grown to 80% confluence in either a control medium or MSC-CM. The plates were scraped using a P200 pipette tip (Cat No. 2789-05-RI, Thermo Fisher, USA) and 3 times rinsed in cell culture media 48 h later. The cells were then treated with either an MSC-CM or a control medium. Under a light microscope (Leica microscope, USA), the wounded regions were captured in 5 randomly selected 100 fields immediately following the wounding (0 h) and after the research (18 h). Analysis was done on the wound's size and its closure.

GIST-882 cells (1 × 10^5^) were seeded in each well of a 6-well plate and grown in MSC-CM for 48 h to conduct a Transwell invasion assay. The GIST-882 cells that had been treated were then collected. The bottom chamber of the top chambers (Cat No. 3401, Corning, NY, USA) was used as a chemoattractant and was seeded with GIST-882 cells suspended in serum-free media. The top chambers were seeded with 1.5 × 10^5^ GIST-882 cells and covered with matrigel (Cat No. 356234, BD Bioscience, USA). The chambers were then incubated for 12 h. Paraformaldehyde was used to fix the membranes, and crystal violet solution was used to stain them. The upper surface of the filter was cleared of cells. Under a microscope (Leica microscope, USA), the invading cells were counted and photographed in at least 6 different fields. Three pictures were processed for each membrane, and each experiment was run in triplicate.

### Cell apoptosis assay

Apoptotic cells were identified using FITC-Annexin V and propidium iodide after GIST-882 cells were grown in control media or MSC-CM for 48 h at 37 °C (Cat No. 556547, BD Biosciences, USA). Briefly, 1 × 10^6^ cells were resuspended in 100 μL of binding buffer with 5 μl of FITC-Annexin V and propidium iodide after being washed with cold PBS. The cells were then incubated for 15 min at room temperature. The number of apoptotic cells was analyzed with a FACScan ( BD FACS Aria™; NJ, USA) and Flow Jo V10 software.

### Cell cycle analysis

100 mm cell culture dishes were planted with 1 × 10^5^ GIST-882 cells per dish. The cells were rinsed 3 times with 10 mL of PBS after being seeded, and then 10 mL of control media or MSC-CM was added to the dish at the 24 h interval. 1 × 10^6^ cells were collected after 48 h and frozen in ice-cold 70% ethanol for 24 h. The cells were then treated in a solution of 200 g/mL RNase A and 10 g/mL propidium iodide. FACS was performed using a BD FACS Aria II SORP (BD Biosciences, Franklin Lakes, NJ, USA). Using the software Modfit, 10,000 events from each trial were counted, and cell cycle patterns were modeled (Verity Software House).

### Cytokine ELISAs

All supernatant samples were acid-activated to measure the TGF- isoform. TGF-β1/2/3 was measured with a commercially available sandwich ELISA kit (Cat No. DB100C/DB250/DY243, R&D Systems, USA) according to the manufacturer's instructions.

### The mRNA expressions of *TGF-β1*, *TGF-β2*, and *TGF-β3*

Total RNAs were extracted for Real-Time qPCR using an ABI Prism 7700. (Applied Biosystems). The TaqMan master mix reagents and the FAM-labeled Taqman probes for human *TGF-1, -2, -3,* SDF-1(CXCL12), VEGF, TNF-α, CXCL16, PDGFA/B/C/D and *GAPDH* (Assays on-demand gene expression products) were purchased from Applied Biosystems and then utilized following the guidelines provided by the manufacturer. Standard curves were determined by repeated tenfold dilutions. RNA samples were evaluated in duplicate for 40 cycles under identical circumstances as those in the same 96-well plates. The sample fluorescence intensities were used to calculate relative fluorescence units by adjusting to the *GAPDH* fluorescence levels. Results are provided as fluorescence relative units.

The *TGF-β1* mRNA forward primer was GAAATT GAGGGCTTTCGCCTTAG, and the *TGF-β1* mRNA reverse primer was GGTAGTGAACCCGTTGAT GTCCA. The *TGF-β2* mRNA forward primer was AAG CCAGAGTGCCTGAACAA, and the *TGF-β2* mRNA reverse primer was GCGCTGGGTTGGAGATGTTA. The *TGF-β3* mRNA forward primer was TGCCAA AGAAATCCATAAATTCGAC, and the *TGF-β3* mRNA reverse primer was AGGTAATTCCTTTAGGGC AGACAGC.

The *SDF1(CXCL12)* mRNA forward primer was CTCAACACTCCAAACTGTGCCC, and the *SDF1(CXCL12)* mRNA reverse primer was CTCCAGGTACTCCTGAATCCAC. The *VEGF* mRNA forward primer was TTGCCTTGCTGCTCTACCTCCA, and the *VEGF* mRNA reverse primer was GATGGCAGTAGCTGCGCTGATA. The *TNF-α* mRNA forward primer was CTCTTCTGCCTGCTGCACTTTG, and the *TNF-α* mRNA reverse primer was ATGGGCTACAGGCTTGTCACTC. The *CXCL16* mRNA forward primer was CCTATGTGCTGTGCAAGAGGAG, and the *CXCL16* mRNA reverse primer was CTGGGCAACATAGAGTCCGTCT. The *PDGFA* mRNA forward primer was CAGCGACTCCTGGAGATAGACT, and the *PDGFA* mRNA reverse primer was CGATGCTTCTCTTCCTCCGAATG. The *PDGFB* mRNA forward primer was GAGATGCTGAGTGACCACTCGA, and the *PDGFB* mRNA reverse primer was GTCATGTTCAGGTCCAACTCGG. The *PDGFC* mRNA forward primer was TGAACCAGGGTTCTGCATCCAC, and the *PDGFC* mRNA reverse primer was TAAGCAGGTCCAGTGGCAAAGC. The *PDGFD* mRNA forward primer was GCGGCTTCACTCTCAGGAGAAT, and the *PDGFD* mRNA reverse primer was CTTGTGTCCACACCATCGTCCT.

### Western blot

Following the manufacturer's instructions, whole cell protein lysates were extracted with ProteoJET Mammalian Cell Lysis Kit (Catalog No. K0301, MBI Fermentas, Canada) supplemented with a full protease inhibitor cocktail. Using a Bradford protein assay kit, protein concentration was measured (Cat No. P0006, Beyotime Institute of Biotechnology, China). On a 12% SDS-PAGE gel, protein samples were separated and then transferred to nitrocellulose membranes (Cat No. HATF00010, Millipore, USA). The membranes were treated overnight at 4 °C with primary antibodies against PI3K, PDK1, AKT, mTOR, p-PI3K, p-PDK1, p-AKT, and p-mTOR TGF-1/2/3 (Abcam, 1:1000) and GAPDH (Abcam, 1:200). (Abcam, 1:1500). Next, secondary antibodies conjugated with horseradish peroxidase were incubated at 4 °C for 30 min. SuperSignal chemiluminescent substrate was utilized to observe protein bands (Thermo Scientific, USA). The GAPDH gene served as the loading control.

### Infection with lentiviral particles

GIST-882 cells were plated and infected on DIV1 with 2.5 MOI of viral particles. The following constructs were used: shTgfbr2 with HPGK-PURO-CMV-TagRFP (Cat No. TRCN0000294600, Sigma, USA).

### Immunofluorescence staining

Immunofluorescence analyses were carried out on paraffin-embedded sections of surgical excision samples obtained from Nanjing Biobank. Prior to embedding, the specimens were fixed in formalin and mildly decalcified overnight in acetic acid. Subsequently, 3 μm-thick sections were cut and stained with anti-CD90 (Cat No. FM049FT, Acris, USA) and CD73 antibodies (Cat No. 551123, Dako, Germany) using the indirect immunoperoxidase-staining technique. The nuclei were counterstained with DAPI. Four sections were selected randomly from each sample for analysis.

### Statistical analysis

All statistical data were expressed as the mean ± standard deviation (SD). The data were analyzed by t-test or ANOVA to determine the statistical significance. *P* < 0.05 was defined to be significant. All statistical analyses were performed using GraphPad Prism (GraphPad Software, Inc., San Diego, Calif.).

The paired-sample t-tests were used to analyze the data of experiments involving 2 groups. For correlation analysis, a two-sided Pearson correlation test was used. Kaplan–Meier survival curves were used to determine survival analysis, and the differences in survival curves were evaluated using the log-rank test. A *P*-value < 0.05 was considered significant.

## Results

### The proportion of MSC in GIST tumor samples correlates with Imatinib resistance and clinical outcome

**Fig. 1:**
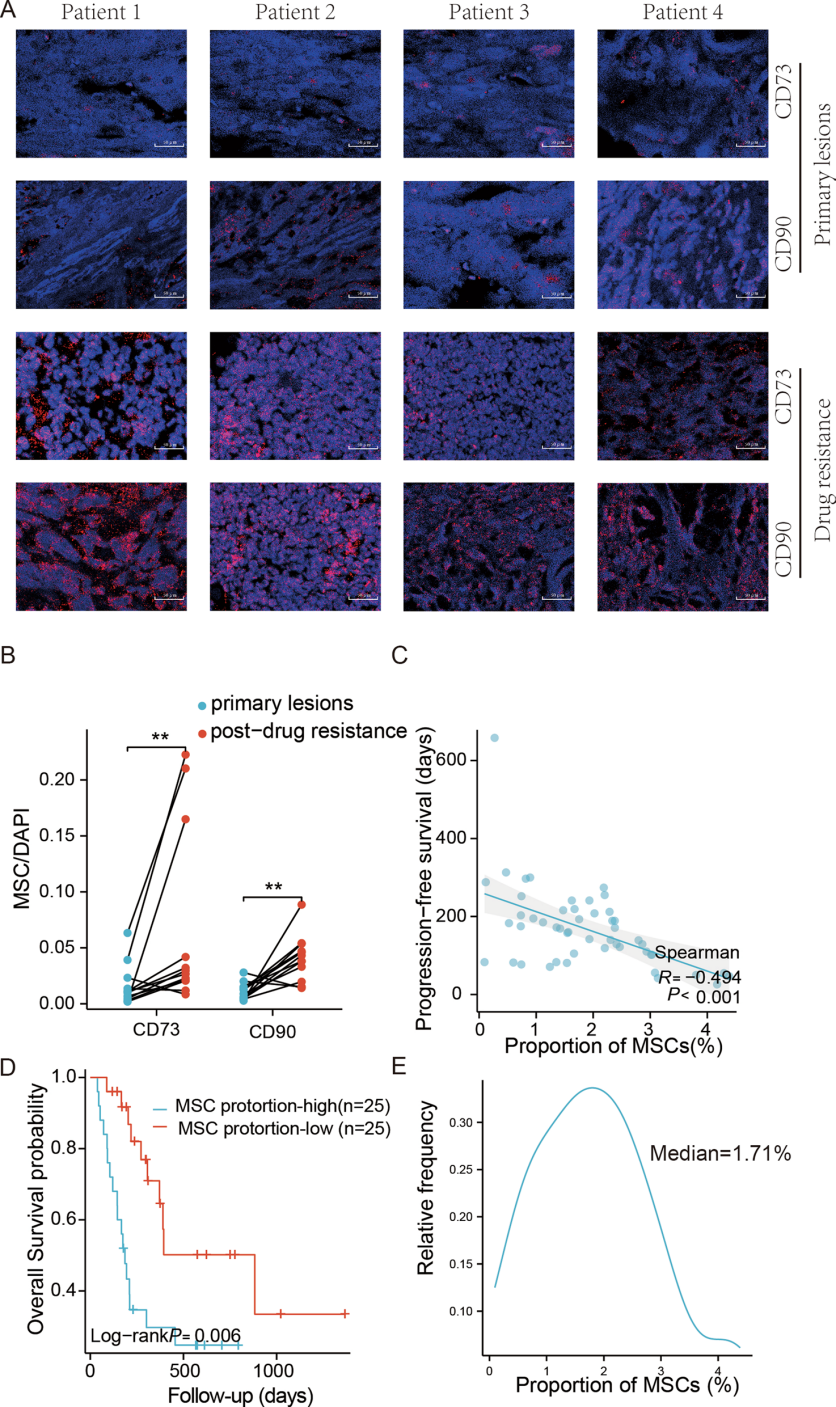
Proportion of MSC in clinical samples correlates with Imatinib resistance and prognosis. Paired samples are drug-sensitive and subsequently drug-resistant surgical samples from the same individual. Immunofluorescence assay for CD73 and CD90 in paired comparative clinical samples (**A**). The relative fluorescence intensity of CD73 or CD90 was significantly enhanced in the drug-resistant group compared to the drug-sensitive group (**B**). The proportion of MSC obtained using the FACS method in the other 50 samples was significantly correlated with progression-free survival (**C**). Overall survival curve analysis was performed by grouping all cases using the median of the proportion of MSC in the 50 samples as the threshold. The proportion of MSC in the tumour tissue was significantly correlated with the overall survival of the patients. The selected threshold is determined to be 1X median, as the data dispersion is minimal and no outliers were detected. Median-1.71% (**D**). Density plot of MSC proportions, The horizontal axis of the graph denotes the MSC proportion across the 50 cases, whereas the vertical axis indicates the probability of occurrence associated with each data point. (**E**) The density plot of the proportion of MSCs amongst 50 cases is graphically represented. The horizontal axis denotes the proportion of MSCs while the vertical axis indicates the frequency of occurrence of each respective proportion. Data are means ± SEM *p < 0.05; **p < 0.01

A total of 12 cases of GIST were enrolled in this study. The clinical information of the patients is displayed in Table 1. Prior to the administration of targeted therapy, all patients exhibited sensitivity to Imatinib. For each patient, pre-Imatinib treatment and post-resistance samples were collected. Fluorescence Immunofluorescence was employed to quantify the proportion of MSC in the specimens. MSC are identified by their positive response to CD73 or CD90 fluorescent antibodies (as illustrated in Fig. 1A). The signal density of MSC was calculated by the ratio of fluorescence intensity of CD90 or CD73 to that of DAPI. The outcomes of the analysis exhibited a significant increase in the density of CD73-positive or CD90-positive MSC cells in the tumor microenvironment following the onset of drug resistance as compared to the corresponding drug-sensitive samples (as demonstrated in Fig. 1B).

**Table 1:** clinicopathologic features in 12 patients with GIST

Characteristic	Primary lesions	Post-drug resistance	p
n	9	9	
T stage, n (%)			0.813
T1	2 (11.1%)	2 (11.1%)	
T2	4 (22.2%)	6 (33.3%)	
T3	2 (11.1%)	1 (5.6%)	
T4	1 (5.6%)	0 (0%)	
M stage, n (%)			0.082
M0	9 (50%)	5 (27.8%)	
M1	0 (0%)	4 (22.2%)	
Pathology, n (%)			0.058
Ib	0 (0%)	1 (5.6%)	
II	5 (27.8%)	4 (22.2%)	
IIIa	3 (16.7%)	0 (0%)	
IIIb	1 (5.6%)	0 (0%)	
IV	0 (0%)	4 (22.2%)	
Gender, n (%)			1.000
Female	3 (16.7%)	3 (16.7%)	
Male	6 (33.3%)	6 (33.3%)	
Mitotic count(< 5/50HPF或 > 10/50HPF), n (%)			1.000
High	7 (38.9%)	7 (38.9%)	
Low	2 (11.1%)	2 (11.1%)	
Tumor site, n (%)			0.156
Mesenterium	0 (0%)	1 (5.6%)	
Omentum	0 (0%)	2 (11.1%)	
Peritoneum	0 (0%)	1 (5.6%)	
Rectum	1 (5.6%)	0 (0%)	
Small intestine	3 (16.7%)	0 (0%)	
Stomach	5 (27.8%)	5 (27.8%)	
Age, mean ± SD	53.78 ± 5.74	58.78 ± 8.18	0.153

**Fig. 2:**
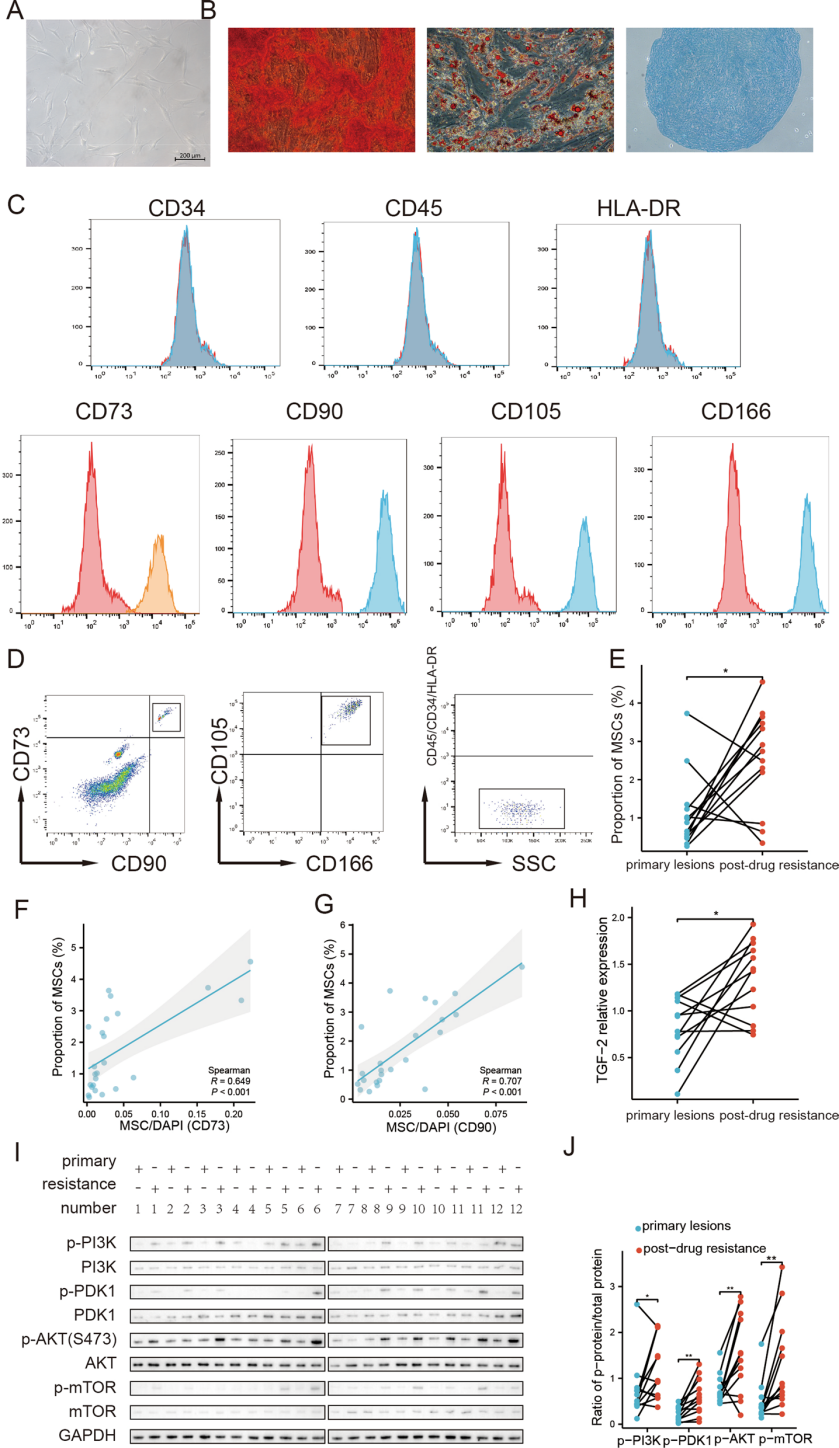
Identification of MSC in clinical samples and variation in TGF-β2/PI3K-AKT. Anti-CD90 positive MACS purified MSC from tumor tissue, and the cells obtained after adherent culture had a typical spindle and stellate morphology (**A**) with the characteristic tri-lineage differentiation ability of MSC. The cells had the ability to differentiate into osteoblasts, adipocytes, and chondrocytes, respectively (**B**). The cells were identified using MSC-related markers, they expressed CD73, CD90, CD105, and CD166 but not CD34, CD45, or HLA-DR (**C**). The FACS method was used to isolate and measure the proportion of MSC in 12 pairs of paired samples. The proportion of MSC was significantly higher in the drug-resistant group compared to the drug-sensitive group (**D**). The proportion of MSC measured by the FACS method and the immunofluorescence method in 12 pairs of paired samples showed consistent trends (**F**, **G**). The transcription of TGF-β2 was significantly higher in the drug-resistant group (**H**). Also, the phosphorylation level of related proteins in the PI3K-AKT pathway was significantly increased in the drug-resistant group (**I**, **J**). Data are means ± SEM *p < 0.05; **p < 0.01

Studies from different research have shown that the expression of the common MSC marker (CD73 or CD90) is also upregulated in gastric cancer cells [15, 16]. We, therefore, conducted a more critical identification of MSC cells in tumor tissue. We collected fresh surgical specimens from GIST patients, isolated cells from them using anti-CD90 MACS, and then identified multiple markers of MSC cells (CD73CD90CD105 positive CD45 negative) and assessed their capacity for tri-lineage differentiation. In line with the definition of MSC, these cells were spindle and stellate morphology (Fig. 2A) and adhered to the plastic dish. After 21 days of growth in the appropriate induction medium, the cells were able to differentiate into osteoblasts, adipocytes, and chondrogenesis respectively (Fig. 2B). These cells all expressed MSC-associated markers CD73, CD90, and CD105 and did not expressCD45 (Fig. 2C). Based on the above results, we prepared the above 24 paired samples into single cell suspensions, which were then separated by FACS and the proportion of MSC(CD73^+^CD90^+^CD105^+^ / CD45^−^) cells to the total cells was calculated. The results showed that the proportion of MSC was significantly increased in samples from the drug-resistant group compared to the drug-sensitive group (Fig. 2E). As the immunofluorescence and FACS methods used for MSC counting in this experiment were deficient in terms of accuracy and sample integrity respectively. We compare the results obtained by these two methods. The results show that there is a significant correlation between the results obtained by the two methods (Fig. 2F, G).

An extra 50 GIST surgical resection samples were picked at random. The proportion of MSC present in tumor samples was determined by means of FACS. Correlation analysis showed that MSC proportion in these samples was negatively correlated with the progression-free survival of the patients (Fig. 1C). Overall survival curves were estimated using the Kaplan–Meier method and differences between groups were assessed using the log-rank test. The median of the proportion of MSC in all 50 samples was used as a threshold for grouping. The P-value indicates significant difference between the two survival curves. (Fig. 1D).

The aforementioned findings indicate a positive correlation between the abundance of MSC within the neoplastic tissue and resistance to Imatinib. Additionally, a higher frequency of MSC was linked with unfavorable prognoses in individuals afflicted with GIST.

### MSC could migrate to GIST tumor cells in vitro and trigger drug resistance

**Fig. 3:**
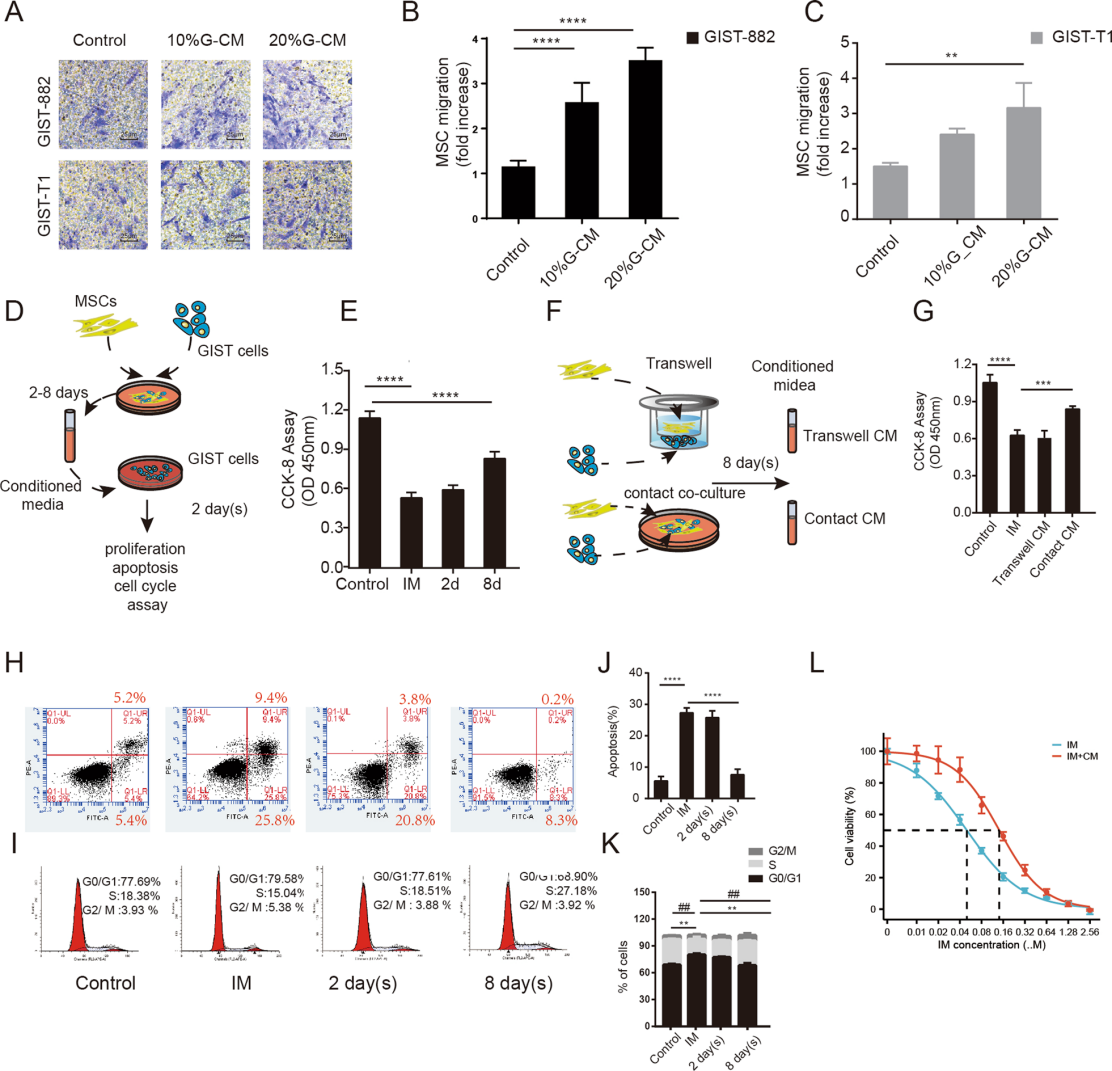
MSC and IM resistance in GIST cells in vitro. Transwell cell migration assay showed that MSC could migrate to GIST-882 and GIST-T1 cells in vitro (**A**–**C**). After mixed co-culture for 2–8 days, CM improves the survival of GIST cells under IM treatment (50 nM) (**D**–**E**). In contrast to the no-contact co-culture of Transwell, MSC can promote drug resistance only when in direct contact with GIST (**F**, **G**). Co-culture with MSC reduced the apoptosis rate of GIST (**H**, **J**) under IM treatment and reduced the proportion of G0/G1 phase cells while increasing the proportion of S phase cells (**I**, **K**). Conditioned medium increases the IC50 of IM on GIST cells (**L**). All experiments were repeated at least thrice. Data are means ± SEM (n = 3 in each group) *p < 0.05; **p < 0.01

Transwell assay results illustrated that human MSC could migrate towards GIST cells in vitro (Fig. 3A–C). To investigate whether MSC could induce resistance to Imatinib in GIST cells in vitro, MSC and GIST were contact co-cultured for 2 or 8 days, and the obtained contact-CM was collected. The CM could enhance the survival of GIST cells under IM treatment, with the effect being particularly significant after 8 days of co-culture (Fig. 2D, E). The half maximal inhibitory concentration (IC50) of Imatinib on GIST-882 cells increased from 49.4 µM to 142.9 µM after the addition of the CM (Fig. 3L).

In contrast, the CM obtained from transwell no-contact co-culture failed to induce drug resistance in comparison to mixed co-culture (Fig. 3F, G). Direct cell-to-cell contact appears to be crucial for creating a milieu that promotes drug resistance. Further analysis revealed that the co-culture of MSC and GIST decreased IM-induced apoptosis of GIST cells (Fig. 3H, J. Treatment with IM led to a significant increase in the proportion of GIST cells in the G0/G1 phase and a decrease in the proportion of cells in the S phase, compared to the control, whereas co-culturing with MSC reversed these effects (Fig. 3I, K). Furthermore, wound healing and transwell assays indicated that the co-culture medium similarly reversed the inhibition of GIST cell migration and invasion by IM (Additional file 1: Fig. S1).

The aforementioned in vitro experiments illustrated that MSC exhibit migratory behavior towards the site of GIST tumors. When MSC were in direct contact with GIST for an extended duration, they generated a conducive environment for augmented resistance to GIST. This alteration in drug resistance may be attributed to the decrease in apoptosis and the shift in the cell cycle of GIST.

### MSC increased TGF-β2 secretion after co-culture with GIST cells

**Fig. 4:**
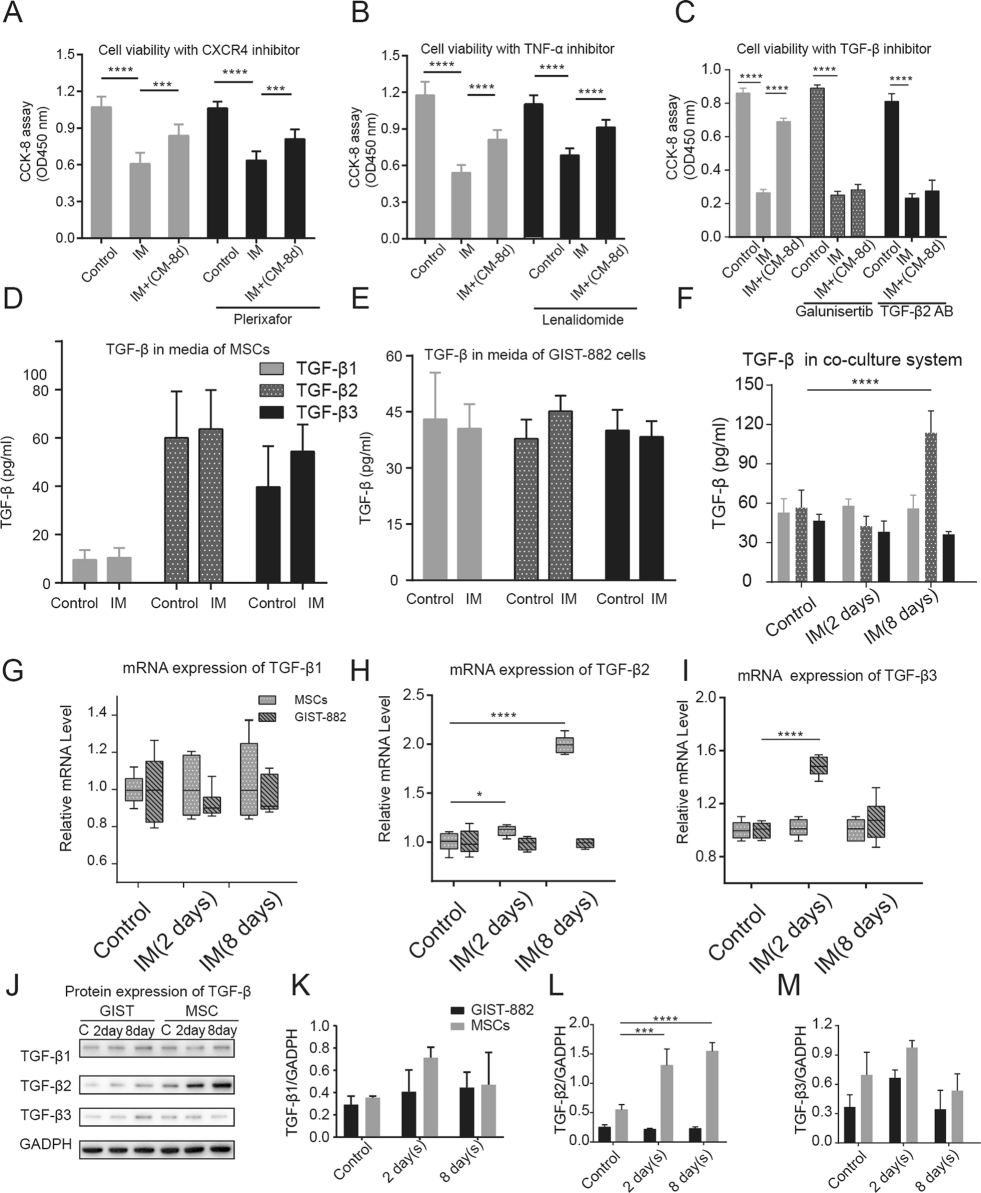
TGF-β2 secreted by MSC may have induced drug resistance in GIST cells. TGF-β inhibitors but not CXCR4 and TNF-α inhibitors antagonize co-culture-induced GIST resistance (**A**–**C**). When cultured alone, there was no significant increase in TGF-β in both MSC or GIST cell media after cultured 8 days. However, there was a significant increase in TGF-β2 in the medium of the co-culture system (**D**–**F**). MSC and GIST cells were isolated after 2–8 days of co-culture, and their mRNA and protein expression were examined. Only the mRNA (**G**–**I**) and protein (**J**–**M**) expression of TGF-β2 was consistently increased in MSC cells. All experiments were repeated at least thrice. Data are means ± SEM (n = 3 in each group) *p < 0.05; **p < 0.01

Multiple investigations have demonstrated that CXCR4, TNF-α, and TGF-β play a role in regulating drug resistance in MSC located in the tumor environment. To explore the underlying mechanism of MSC-mediated GIST drug resistance, Plerixafor, Lenalidomide, and Galunisertib were introduced into the co-culture system as CXCR4, TNF-α, and TGF-β inhibitors, respectively, to evaluate their impact on GIST cell survival. The findings revealed that only the TGF-β inhibitor could counteract the protective effect of the conditioned medium on GIST cells following IM treatment (Fig. 4A–C).

To further validate whether co-culture induces an upsurge in TGF-β expression, we measured the levels of TGF-β in the medium of MSC and GIST cells, cultured independently and mixed co-cultures after incubation with Imatinib for the same duration, using ELISA. The findings demonstrated a significant rise in the content of TGF-β2 in the medium of the co-culture system after an 8-day co-culture period (Fig. 4F). Conversely, the contents of all three primary isoforms of TGF-β remained unchanged when cultured independently (Fig. 4D, E).

Following the co-culture, we isolated MSC and GIST cells using magnetic beads and measured their mRNA transcription and protein expression of TGF-β, respectively. The results indicate a consistent increase in the mRNA and protein expression of TGF-β2 in MSC following 2 and 8 days of co-culture, as illustrated in Fig. 4G, H, I and Fig. 4G, K, L, M respectively. These findings suggest that the secretion of TGF-β2 by MSC through paracrine signaling may trigger GIST resistance.

### MSC trigger drug resistance in GIST cells via the TGF-β2-PI3K-AKT signaling pathway

**Fig. 5:**
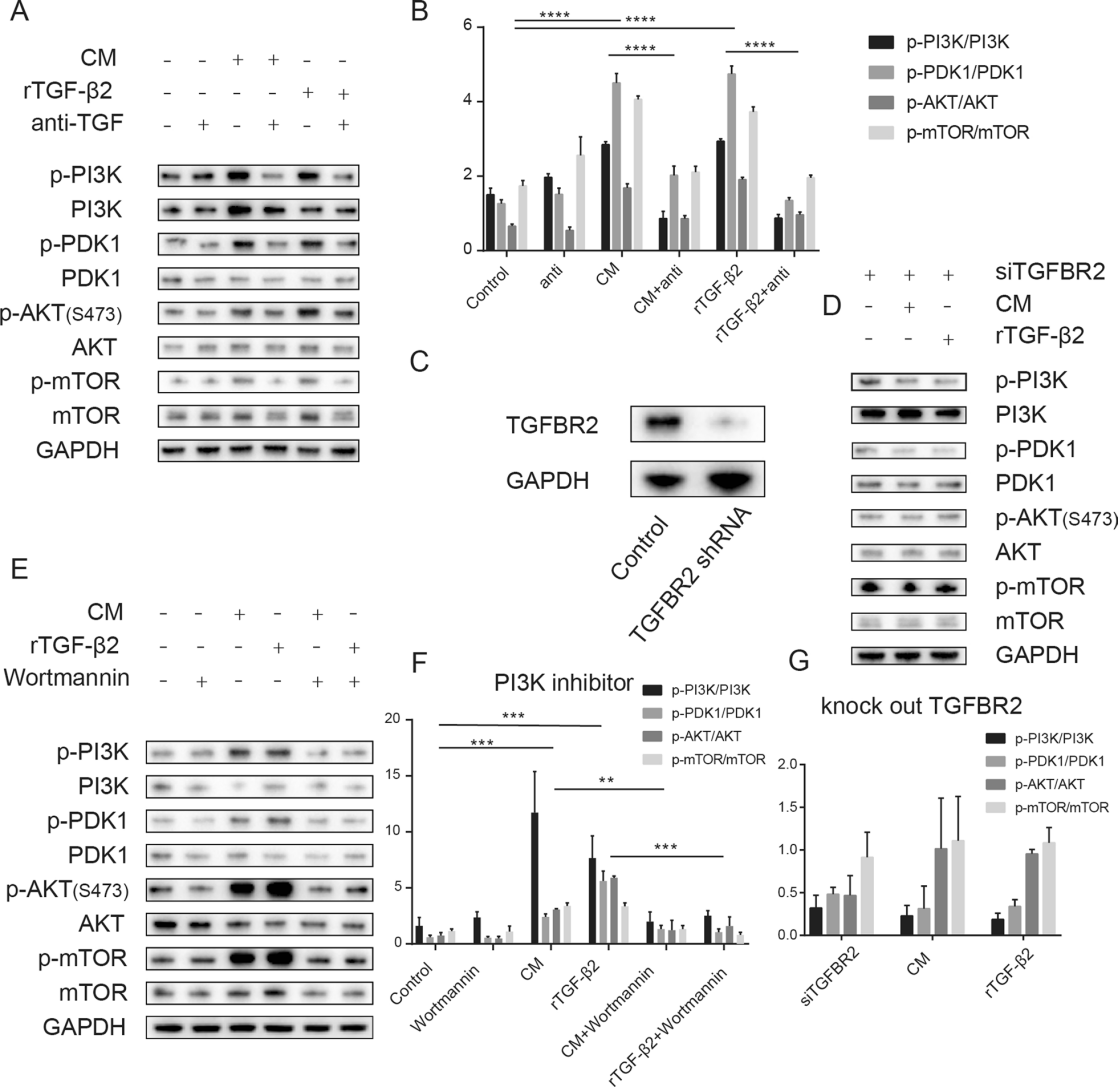
MSC-secreted TGF-β2 activates the PI3K-AKT signaling pathway in GIST cells. Both conditioned medium and recombinant TGF-β2 protein activate the PI3K-AKT signaling pathway of GIST, and the TGF-β2 monoclonal antibody antagonizes these effects (**A**, **B**). After silencing receptor II in the TGF-β receptor complex on the surface of GIST cells (**C**), both conditioned medium and recombinant TGF-β2 protein failed to activate the PI3K-AKT pathway of GIST (**D**, **G**). The PI3K-AKT signaling pathway inhibitor Wortmannin was able to antagonize the activation of the PI3K-AKT signaling pathway by conditioned medium and recombinant TGF-β2 protein (**E**, **F**). All experiments were repeated at least thrice. Data are means ± SEM (n = 3 in each group) *p < 0.05; **p < 0.01

Numerous investigations have demonstrated that downstream signaling of TGF-β2 is extensively connected with the PI3K-AKT signaling pathway. Notably, PI3K-AKT represents one of the downstream signals of GIST KIT/PDGFRA, which is implicated in apoptosis and cell cycle regulation. In order to further scrutinize the mechanism behind TGF-β2-induced GIST drug resistance, we examined the impact of contact-CM on the PI3K-AKT signaling pathway of GIST. Our findings showed that both contact-CM and recombinant TGF-β2 protein provoked the activation of the PI3K-AKT signaling pathway of GIST and phosphorylated its downstream proteins. However, when a TGF-β2 monoclonal antibody was introduced into the conditioned medium, blocking the TGF-β2 signal, this effect was antagonized (Fig. 5A, B).

To validate the impact of TGF-β on GIST drug resistance, we proceeded to silence TGFBR2 in GIST cells, which comprises the TGF-β receptor complex located on the cell membrane surface (Fig. 5C). Upon TGFBR2 silencing, neither the conditioned medium nor recombinant TGF-β2 protein triggered the activation of the PI3K-AKT signaling pathway in GIST cells (Fig. 5D, G).

Upon treating GIST cells with Wortmannin, an inhibitor of the PI3K-AKT signaling pathway, both the conditioned medium and recombinant TGF-β2 protein were unable to activate the PI3K-AKT signaling pathway in GIST cells (Fig. 5E, F).

**Fig. 6:**
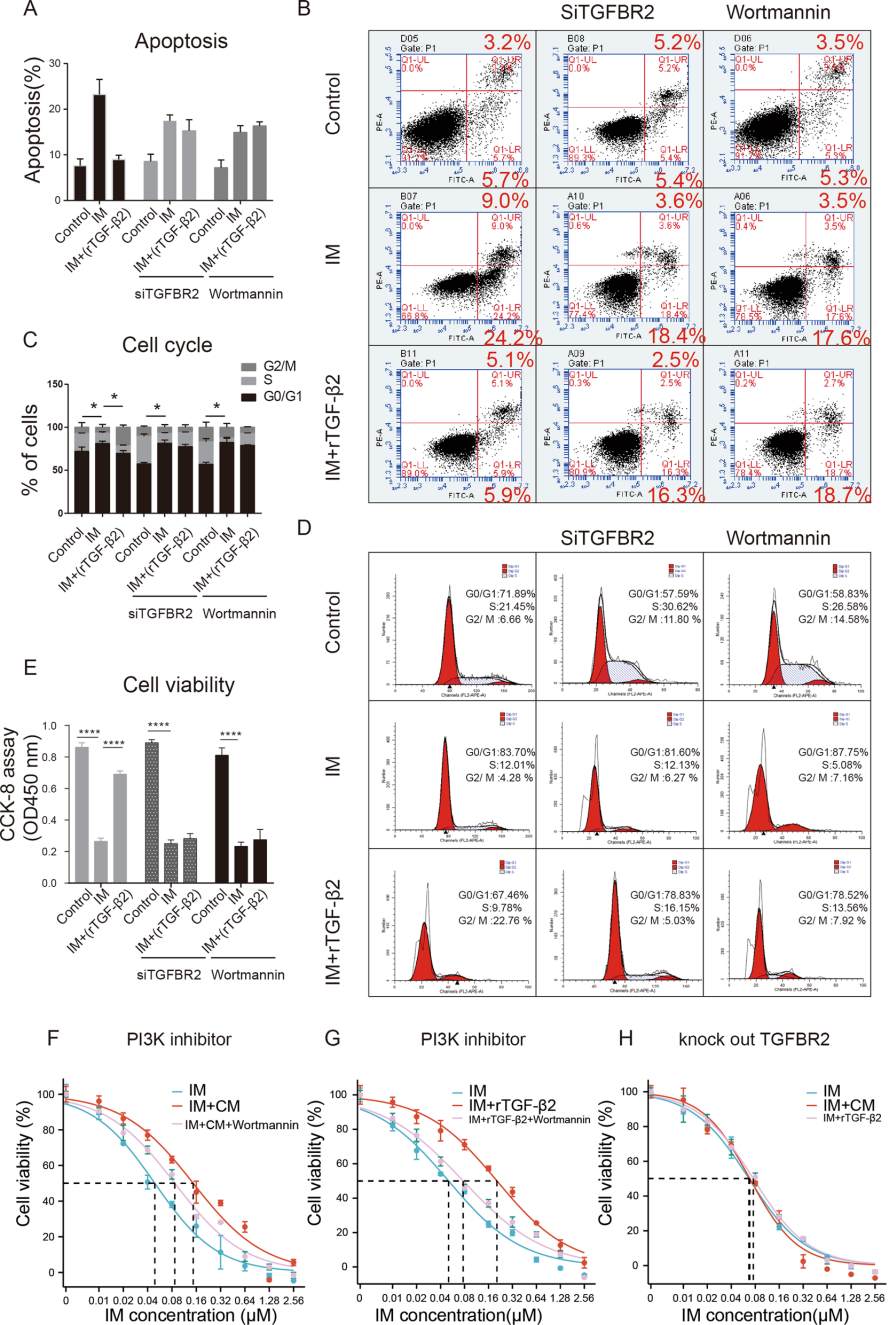
MSC activates the PI3K-AKT signaling pathway via TGF-β2 to promote drug resistance in GIST cells. Flow cytometry experiments showed that recombinant TGF-β2 reduced apoptosis in IM-treated GIST cells (**A**, **B**) and reduced G0/G1 phase cell ratio (**C**, **D**). Silencing of the TGF-β receptor and PI3K-AKT inhibitor antagonized this effect (**A**–**D**). CCK-8 assay showed that recombinant TGF-β2 increased the survival of GIST cells under IM treatment, and silencing of the TGF-β receptor and PI3K-AKT inhibitor antagonized this effect (**E**). Conditioned medium (CM) for MSC co-culture with GIST cells can partially restore the IC50 of IM inhibition of GIST proliferation (**F**). And recombinant TGF-β2 has similar effects to CM. But the addition of PI3K inhibitors Wortmannin counteracted this effect (**G**). After silencing the TGF- receptor on GIST cells, neither CM nor recombinant TGF-2 could increase the IC50 of IM to inhibit the growth of GIST cells anymore (**H**). All experiments were repeated at least thrice. Data are means ± SEM (n = 3 in each group) *p < 0.05; **p < 0.01

Recombinant TGF-β2 protein treatment led to a decrease in the rate of GIST apoptosis (as portrayed in A, B), a deviation from their normal cell cycle progression (as demonstrated in Fig. 6C, D), and an elevation in their survival rate (as exemplified in Fig. 6E) upon being subjected to IM treatment. In contrast, the inhibition of TGFBR2 or the use of the PI3K inhibitor could neutralize these effects, as demonstrated by the data presented in Fig. 6A–C.

Concomitantly, the impact of the aforementioned interventions on GIST cell growth inhibition by IM was assessed. The findings indicate that the addition of CM can raise the IC50 of IM from 55.2 µM to 155.3 µM, which could be restored to 107.1 µM by administering a PI3K inhibitor, as shown in Fig. 6F. The introduction of recombinant TGF-β2 to the culture medium mitigated the inhibitory effect of IM, akin to the addition of conditioned medium (CM), eliciting a rise in the IC50 of IM from 51.5 µM to 264.4 µM. However, subsequent administration of PI3K inhibitor restored the IC50 to 79.8 µM (Fig. 6 G). In contrast, silencing TGFBR2 in GIST cells had no significant effect on either CM or recombinant TGF-β2 mediated inhibition of IM (Fig. 6H).

### Transcription of TGF-β2 and activation of PI3K-AKT pathway correlate with imatinib resistance and clinical prognosis in clinical samples

We scrutinized the TGF-2 and PI3K-AKT signaling pathways in 12 paired clinical samples of GIST. Our findings revealed a significant rise in the TGF-2 transcripts in the post-treatment samples when juxtaposed with their pre-treatment counterparts (Fig. 2E). Additionally, the levels of phosphorylated PI3K-AKT-related proteins were observed to be significantly elevated (Fig. 2I, J). The results presented above imply that the in vivo MSC might additionally promote GIST resistance via the activation of the PI3K-AKT pathway through the paracrine action of TGF-β2.

**Fig. 7:**
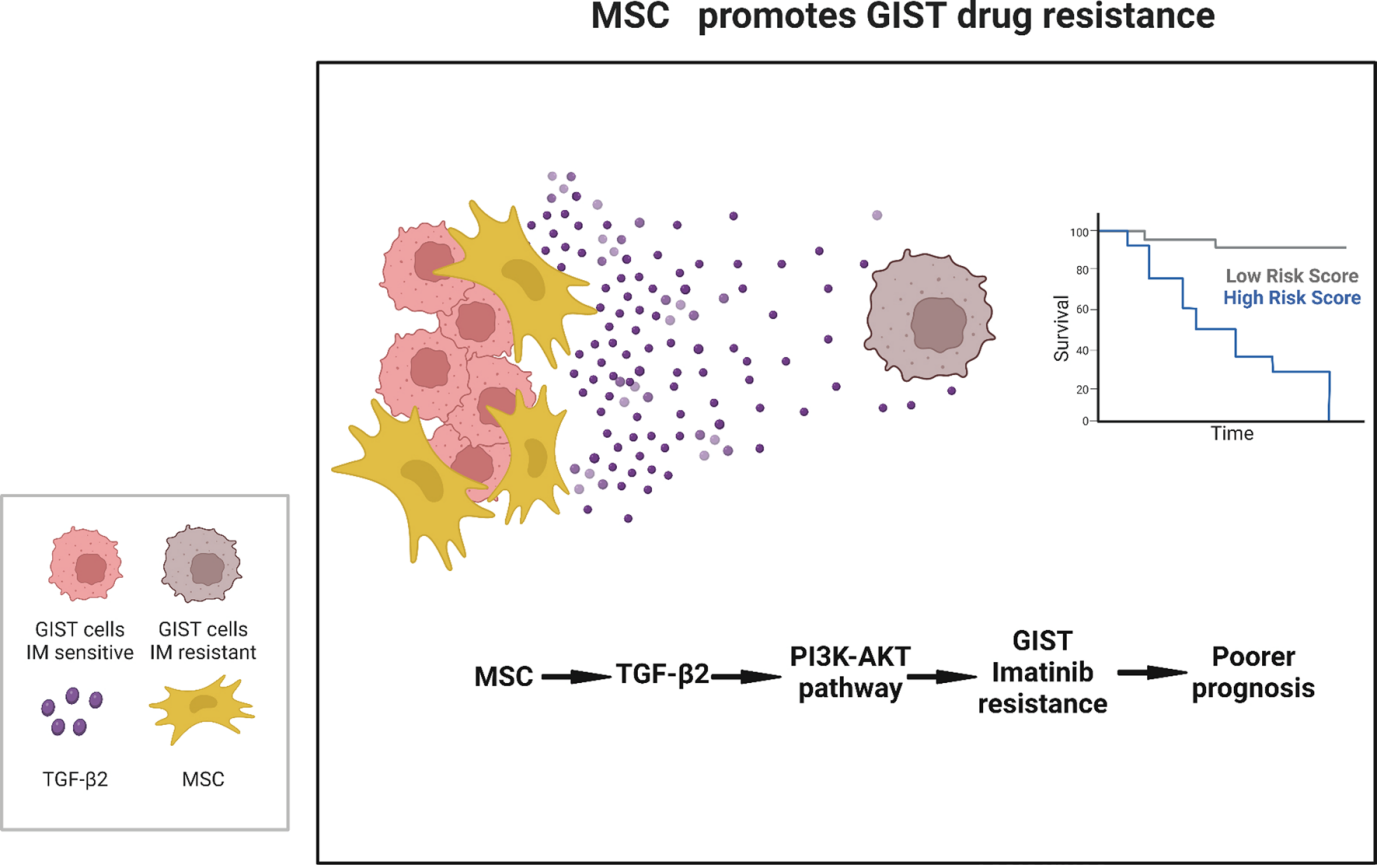
Proposed model of MSC-induced GIST resistance. MSC present in the tumor microenvironment induce resistance to Imatinib in GIST. This resistance is mediated through activation of the PI3K-AKT-mTOR pathway in tumor cells by TGF-β2 secreted by the mesenchymal stem cells. The quantity of MSC within the tumor tissue is correlated with both drug resistance and prognosis. Created with BioRender.com

Taken together, the data suggest that MSC may induce tumor resistance via a paracrine pathway involving TGF-β2 activation of the PI3K-AKT signaling cascade in GIST cells (Fig. 7).

## Discussion

Despite the significant improvements in patient survival brought about by targeted therapies such as Imatinib, drug resistance remains an issue that patients with GIST must confront. Research has indicated that primary drug resistance can develop within six months and is linked to PDGFRA p.D842V substitution or KIT exon 9 duplications. Furthermore, secondary mutations in KIT/PDGFRA that arise after treatment can lead to secondary drug resistance [6, 17, 18]. Nonetheless, a subset of patients who exhibit drug resistance does not harbor the aforementioned mutations that have established significance. Thus, further scrutiny of drug resistance mechanisms can facilitate the development of more efficacious countermeasures. Extensive research has established that the emergence of drug resistance in several tumor types is linked to MSC present in the tumor microenvironment. Clinical investigations have also demonstrated that MSC in the GIST tumor microenvironment are associated with the onset of drug resistance and a poor prognosis.

MSC have been recognized as a significant constituent of tumor stroma, exhibiting both pro-tumorigenic and anti-tumorigenic effects [10, 19]. TGF-β frequently assumes a crucial function in the interplay between MSC and tumors [20, 21] and exhibits intricate regulatory effects on both MSC and tumor cells and plays a crucial role in their interaction. Moreover, the three major isoforms of TGF-β are known to have distinct biological functions in vivo [22]. Studies have demonstrated that TGF-β1 is predominantly secreted by human MSC [23–27]. Our own investigations have revealed that MSC can stimulate GIST drug resistance, an effect that can be countered through the use of TGF-β inhibitors or the silencing of TGF-β receptors on GIST cells.

According to our findings, MSC are capable of inducing drug resistance in GIST cells via the secretion of TGF-β2. Notably, the expression of TGF-β2 by MSC did not exhibit significant changes when cultured alone or in non-contact co-culture conditions. Furthermore, short-term co-culture of MSC with GIST cells did not result in a substantial alteration of TGF-β2 expression. It was only after 8 days of contact co-culture that a significant increase in TGF-β2 expression by MSC was observed, indicating that MSC have the ability to promote tumor resistance solely after the phenotypic modulation of the tumor microenvironment has occurred. What is the underlying mechanism responsible for this phenotypic transition? Numerous studies have indicated that MSC contribute to the tumor-permissive microenvironment by either differentiating into cancer-associated fibroblasts (CAFs) or activating existing CAFs. A recent study demonstrated that TGF-β1 promotes the transformation of resident fibroblasts into CAFs in GIST. These CAFs promote cell migration in vitro and metastasis in vivo [28]. It has been demonstrated that MSC can differentiate into CAFs over the course of a few days to several weeks in vitro [29]. Further investigation is required to elucidate the specific alterations that occur in MSC within the tumor microenvironment, which confer a drug-resistant phenotype. It remains to be determined whether these alterations are linked to the differentiation of MSC into CAFs.

Tumor tissue can activate TGF-β secretion by MSC through various mechanisms. Breast cancer cells have been shown to induce the secretion of TGF-β by MSC through direct cell–cell contact [30]. And pancreatic cancer cells can induce the differentiation of MSC into cancer-associated fibroblasts (CAFs) through the secretion of matrix metalloproteinases (MMPs), which can remodel the ECM and activate TGF-β secretion [31]. In addition, it has been studied that Tumor cells can secrete cytokines and growth factors such as IL-6 or SDF-1 that can activate TGF-β secretion by MSC [32, 33]. Other studies have shown that tumor cells that interact with MSC via ligand receptors include SDF-1, VEGF, TNF-α, PDGF, and CXCL16 [34–36]. We have examined the transcription of the above ligands in GIST cells in the co-culture system. The findings revealed an augmentation in SDF-1 transcriptional expression in GIST cells following contact co-culture (Additional file 1: Fig. S3), which is likely to underlie the promotion of phenotypic transition in MSC and the secretion of TGF-β2.

In light of our findings, both TGF-β and SDF-1 may contribute to the mechanisms underlying alterations in MSC phenotype. Furthermore, exploring the implication of exosomes and inflammatory mediators, including prostaglandin E2, interleukin-4, and interleukin-10, in the reciprocal communication between mesenchymal stem cells and gastrointestinal stromal tumor cells within co-culture systems constitutes a matter of profound scientific interest.

GIST is characterized by constitutive activating mutations of *KIT/PDGFRA*. And PI3K-AKT signaling is one of the downstream signaling pathways of KIT/PDGFRA [37]. PI3K-AKT can also be activated by TGF-β and participate in cell proliferation and apoptosis regulation. In our study, Conditioned medium or recombinant TGF-β2 protein promoted drug resistance while activating the PI3K-AKT signaling pathway. Either TGF-β2 monoclonal antibody or silencing the TGF-β receptor in GIST could inhibit this promotion. The use of PI3K-AKT inhibitor, meanwhile, antagonized the promotion of drug resistance by TGF-β2. This result suggested that TGF-β2 could promote GIST growth by activating the PI3K-AKT signaling pathway, a signal downstream of KIT/PDGFRA, thereby circumventing the direct inhibition of KIT/PDGFRA activity by targeted therapy.

Given that TGF-β/PI3K-AKT-mTOR signaling may play a pivotal role in inducing drug resistance in GIST, targeting this pathway represents a novel approach to mitigate TKI resistance. It has been demonstrated that the mTOR inhibitor rapamycin can activate the TGF-β receptor via a mechanism independent of TGF-β, while maintaining its inhibitory effect on mTOR, ultimately leading to the suppression of tumor cell proliferation [38]. This characteristic renders rapamycin a potentially suitable therapeutic agent for targeting the GIST resistance pathway elicited by MSC. On one hand, it competes with the TGF-β receptor, while on the other, it suppresses the downstream mTOR signaling cascade. Several studies have suggested that while rapamycin exerts a suppressive effect on tumor growth, its administration at high concentrations may elicit unacceptable side effects, while low concentrations are insufficient to impede tumor progression [39]. If low doses of rapamycin can effectively counteract GIST resistance, a novel therapeutic approach may involve combining rapamycin with Imatinib.

The MSC utilized in the in vitro experiments of this investigation were procured from the bone marrow of healthy individuals. It has been reported that the physiological condition of MSC donors can influence their physiological operations, including the generation of TGF-β. Therefore, further studies are necessary to compare the impact of bone marrow MSC and adipose tissue MSC on drug resistance development in GIST patients, and to further authenticate our findings in vivo.

Based on the latest research, it is worth considering the relevance of cytokines, hypoxia, and 3D culture to the tumor microenvironment in comparison to the co-culture models. Exploring the effects of these factors in the co-culture models may provide insights into their potential impact on the sensitivity of Imatinib in both contact and non-contact models. Additionally, further investigation into the discrimination approach between the two co-cultured cells could help elucidate the underlying mechanisms driving the observed effects.

## Conclusion

In summary, this study points to the capability of MSC to promote GIST resistance in targeted therapies. This effect is achieved by paracrine TGF-β2 activating the PI3K-AKT-mTOR pathway, affecting further tumor cell apoptosis and cell cycle. The findings above suggest a novel mechanism of drug resistance involving the activation of paracrine pathways by interaction with MSC in the tumor microenvironment. It also presents a new target for treating patients with drug-free mutations and a measurable indicator that can help predict prognosis (Fig. 7). The primary images of all western blots can be found in Additional file 2.

## Supplementary Information

**Additional file 1: Figure S1.** MG-CM enhanced migration and invasion of GIST cells treated with Imatinib. **Figure. S2. **In-depth characterisation of BM-MSC cells. **Figure S3. **Changes in the expression of ligands on GIST-882 cells after co-culture.

**Additional file 2.** Original gel picture.

## Data Availability

The data set used and/or analyzed during the current study is available from the corresponding author upon reasonable request.

## Abbreviations

TGF-β: Transforming Growth Factor Beta
IM: Imatinib
MSC: Mesenchymal stem cell
GIST: Gastrointestinal stromal tumors
TKI: Tyrosine kinase inhibitors
TGF-β: Transforming Growth Factor Beta
PDGFRA: Platelet-derived growth factor receptor-α
TME: The tumor microenvironment
BMMSC: Bone marrow derived MSC
CM: Conditioned medium
