# Supplementary material for: Mesenchymal stromal cells promote the drug resistance of gastrointestinal stromal tumors by activating the PI3K-AKT pathway via TGF-β2
Source: J Transl Med. 2023 Mar 25;21:219. doi: 10.1186/s12967-023-04063-0 (PMC10040136; doi:10.1186/s12967-023-04063-0)
Supplement: Supplementary file 1 — Additional file 1: Figure S1. MG-CM enhanced migration and invasion of GIST cells treated with Imatinib. Figure. S2. In-depth characterisation of BM-MSC cells. Figure S3. Changes in the expression of ligands on GIST-882 cells after co-culture. [file 12967_2023_4063_MOESM1_ESM.docx]

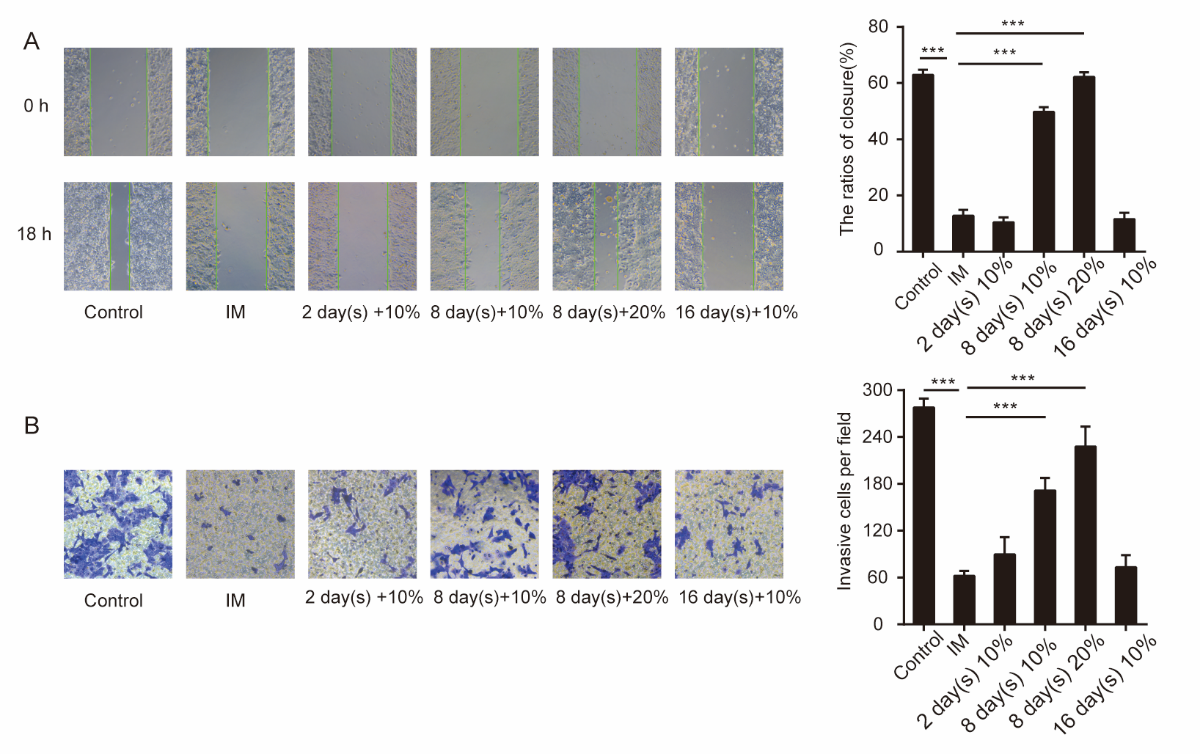


Additional file 1: Fig. S1. MG-CM enhanced migration and invasion of GIST cells treated with Imatinib.

(A)(C) Wound-healing assay shows that CM (only co-cultured for 8 days) dose-dependently promoted the migration of GIST-882 cells. (***P<.001)

(B)(D) Transwell invasion assay shows that MG-CM (only co-cultured for 8 days) dose-dependently promoted the invasion of GIST-882 cells. (***P<.001)

The results are presented as mean±standard deviation; ***P<0.001. MSCs, mesenchymal stem cells, MG-CM, MSCs, and GIST cells co-culture conditioned media.


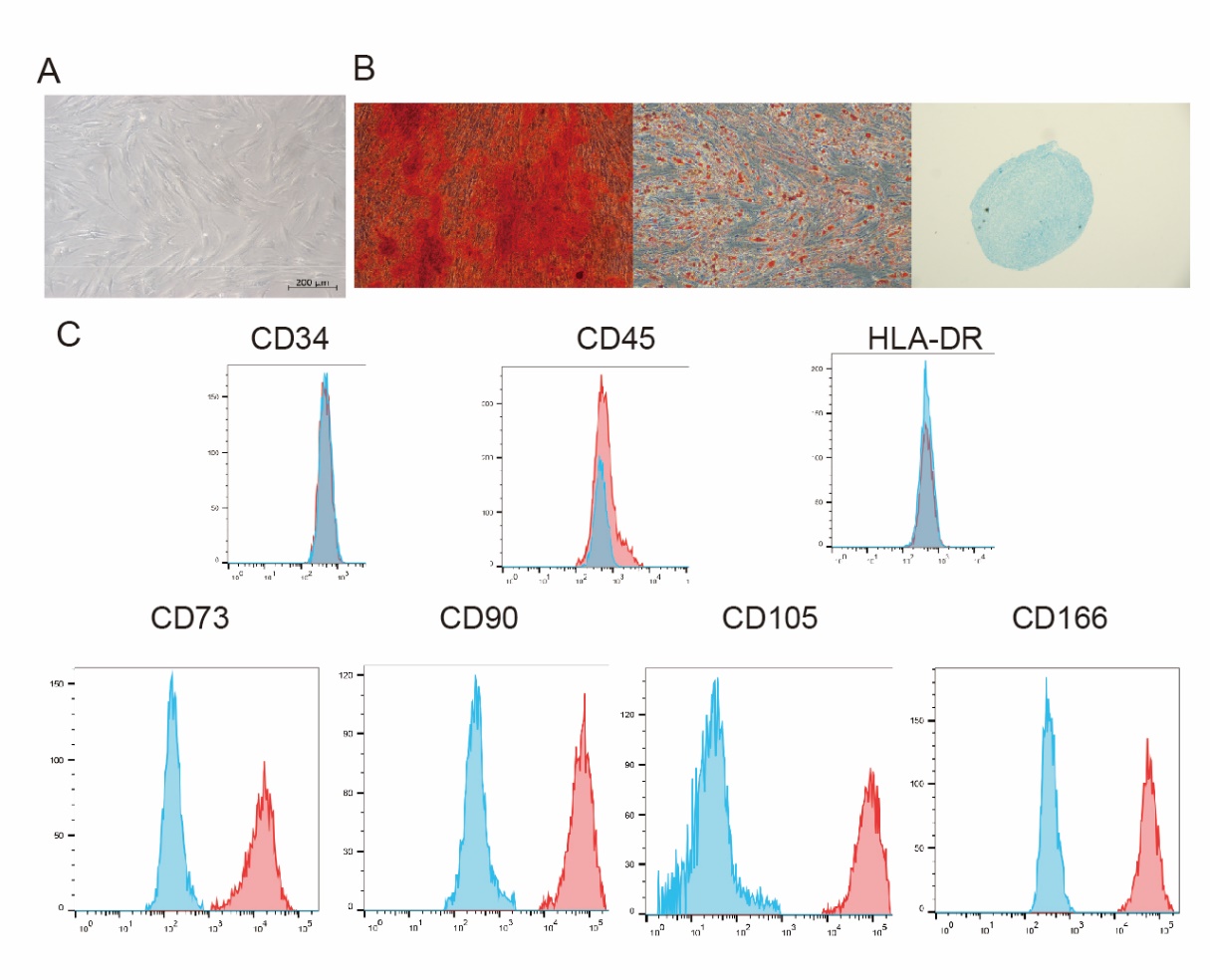


Additional file 1: Fig. S**2. In-depth characterisation of BM-MSC cells**

Anti-CD90 positive MACS purified MSCs from tumor tissue, and the cells obtained after adherent culture had a typical spindle and stellate morphology (A) with the characteristic tri-lineage differentiation ability of MSCs. The cells had the ability to differentiate into osteoblasts, adipocytes and chondrocytes, respectively (B). The cells were identified using MSC-related markers, they expressed CD73, CD90, CD105 and CD166 but not CD34,CD45 or HLA-DR (C)


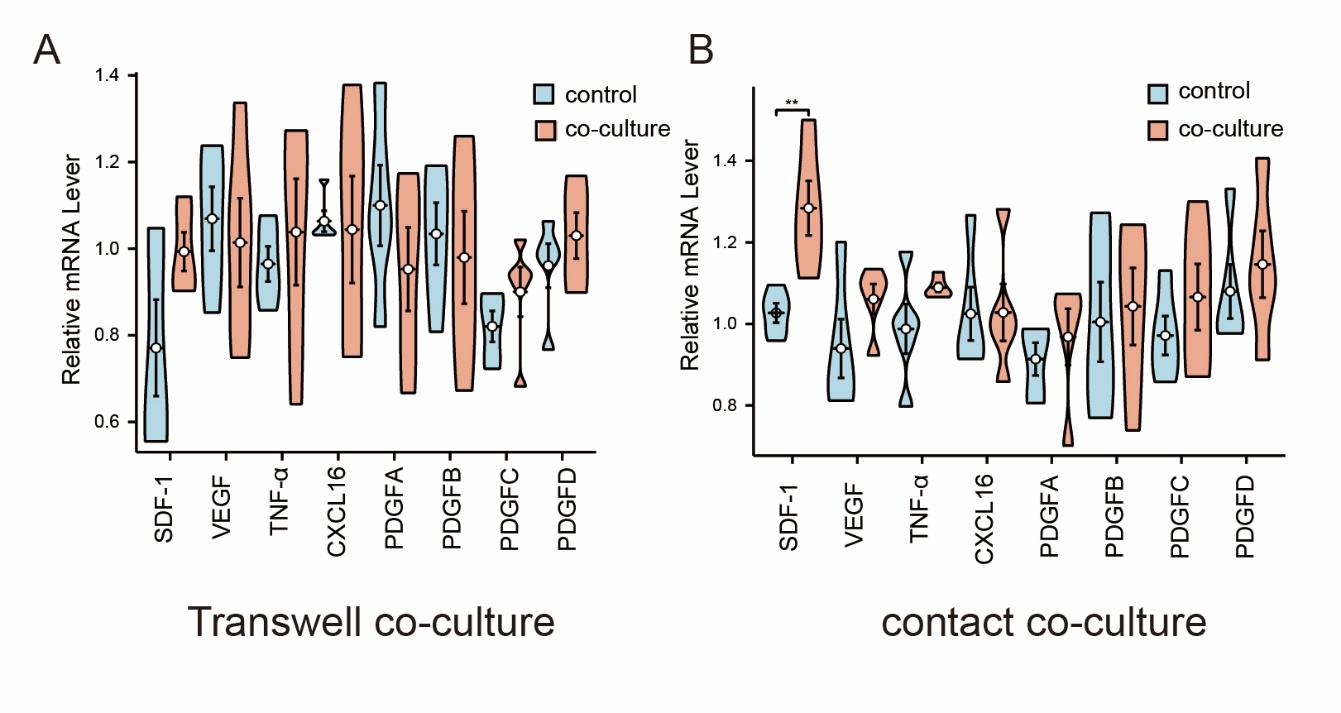
 Additional file 1: Fig. S**3. Changes in the expression of ligands on GIST-882 cells after co-culture**

Alterations in the transcriptional regulation of SDF-1, VEGF, TNF-α, PDGF, and CXCL-16 genes are known to impact the phenotypic characteristics of mesenchymal stem cells (MSCs) in the context of tumor tissue, as evidenced by existing literature. GIST-882 cells were subjected to non-contact co-culture with mesenchymal stem cells (MSCs) in vitro(A). GIST-882 cells are co-cultured with MSC cells in a direct, mixed co-culture(B).
